# Risk factors for pituitary apoplexy: a meta-analysis and development of a clinical prediction nomogram

**DOI:** 10.3389/fneur.2026.1772791

**Published:** 2026-03-10

**Authors:** Haipeng Chen, Ning Huang, Rui Tang, Jin Chen, Guanjian Zhao

**Affiliations:** Department of Neurosurgery, The Second Affiliated Hospital of Chongqing, Medical University, Chongqing, China

**Keywords:** meta-analysis, nomogram, pituitary adenoma, pituitary apoplexy, risk prediction

## Abstract

**Purpose:**

This study aimed to identify significant risk factors for pituitary apoplexy in patients with pituitary adenomas through a meta-analysis and to develop an individualized nomogram for clinical decision-making.

**Methods:**

A two-part investigation was conducted. First, a meta-analysis of published studies identified risk factors for pituitary apoplexy and calculated pooled odds ratios (ORs) and 95% confidence intervals (CIs). Second, a retrospective cohort of 234 patients was used to construct and validate a nomogram based on multivariate logistic regression.

**Results:**

The meta-analysis included six studies, revealing that non-functioning pituitary adenomas (OR = 1.93, 95% CI: 1.38–2.70), male sex (OR = 2.57, 95% CI: 1.85–3.58), and hypertension (OR = 2.53, 95% CI: 1.54–4.15) were significantly associated with pituitary apoplexy. The nomogram demonstrated excellent predictive performance with AUCs of 0.86 in the training set and 0.83 in the validation set. Calibration curves showed good agreement between predicted and observed probabilities. The Hosmer–Lemeshow test yielded P values of 1 and 0.272 in the training and validation cohorts, respectively. Decision curve analysis demonstrated significant net clinical benefit in both cohorts.

**Conclusion:**

This study identified key predictors of pituitary apoplexy and developed a nomogram that may help stratify risk and guide preventive and therapeutic strategies.

## 1 Introduction

Pituitary apoplexy (PA) is an acute clinical syndrome resulting from hemorrhage and/or infarction within a pituitary adenoma. It typically presents with sudden-onset headache, visual disturbances, ophthalmoplegia, and altered consciousness (1, 2). According to reports, the annual prevalence is approximately 62 cases per 1,000,000 population, and the annual incidence is about 1.7 cases per 1,000,000 people. It occurs in approximately 2% to 12% of patients with various types of pituitary adenomas (2). Although PA is relatively rare, its abrupt onset and severe progression can lead to permanent vision loss, neurological deficits, endocrine crises, and even death, resulting in poor prognosis. Furthermore, the high disability rate and significant medical dependence associated with PA not only impose a substantial financial burden on patients but also place increased pressure on healthcare systems.

However, existing research on pituitary adenoma-associated PA remains limited, and the findings from previous studies vary significantly (3). Currently, there is no standardized criterion for the initial diagnosis of PA (4), and these discrepancies may stem from differences in study methodologies, sample sizes, and inclusion criteria (5). Although previous studies have explored the potential mechanisms underlying PA, a clear consensus has yet to be established.

Current studies suggest that tumor growth exceeding its blood supply can lead to ischemic necrosis. Additionally, various factors have been identified as potential triggers for PA, including antiplatelet and anticoagulant therapy, dopamine agonists, gonadotropin-releasing hormone agonists, previous surgical history, head trauma, endocrinological stimulation tests, and systemic diseases (2, 6–8).

A thorough investigation of these risk factors is crucial for the early diagnosis, prevention, and intervention of PA. However, there is still a lack of systematic integration and high-quality risk prediction tools. In recent years, nomograms have been increasingly utilized for disease risk prediction, demonstrating strong predictive performance across various patient populations (9–11).

In this study, we aimed to systematically investigate the risk factors for PA using a two-step approach. First, we performed a meta-analysis of relevant studies to identify statistically significant risk factors. Second, we developed and validated a nomogram based on clinical data from our center to create a reliable predictive tool. This combined approach is intended to provide robust evidence for risk assessment and support clinical decision-making for patients with pituitary adenomas.

## 2 Methods

### 2.1 Meta-analysis

#### 2.1.1 Search strategy

We conducted a systematic search of PubMed, Embase, the Cochrane Library, and Web of Science to identify relevant studies published from database inception to January 8, 2025. The search was performed using the following keyword combination: “pituitary adenoma” AND (“apoplexy” OR “hemorrhage” OR “infarction”). Two independent researchers screened all eligible articles, and any discrepancies were resolved through consensus.

#### 2.1.2 Selection criteria

Inclusion Criteria: (1) Clinical case-control studies involving patients with pituitary adenoma and PA; (2) Studies reporting risk factors associated with PA; (3) Studies providing multivariable analysis results, including odds ratio (OR) or relative risk (RR) with 95% confidence intervals (CI).

Exclusion Criteria: (1) Reviews, meta-analyses, case reports, or basic science studies; (2) Studies reporting only unadjusted univariate analysis; (3) Studies with incomplete or flawed data.

Finally, eligible studies were selected by screening titles and abstracts, followed by a full-text review to confirm inclusion.

#### 2.1.3 Data extraction and quality assessment

Two reviewers independently extracted data, including study characteristics (author, year, country, sample size, study design) and reported outcomes. Study quality was assessed using the Newcastle-Ottawa Scale (NOS), with scores categorized as low (0–3), moderate (4–6), or high quality (7–9, 12). Disagreements were resolved through discussion.

#### 2.1.4 Statistical analysis

Meta-analysis was performed using Review Manager (version 5.3.5) and Stata (version 14.0). Pooled ORs with 95% CIs were calculated to estimate associations between risk factors and PA. Heterogeneity was assessed using the *I*^2^ statistic and Q-test: *I*^2^ > 50% or *P* < 0.10 indicated substantial heterogeneity, prompting use of a random-effects model. Otherwise, a fixed-effects model was applied. A two-sided *P*-value < 0.05 was considered statistically significant.

### 2.2 Nomogram model

#### 2.2.1 Patient selection

Clinical data were collected from patients diagnosed with pituitary adenomas and PA who underwent surgical treatment at the Department of Neurosurgery, Second Affiliated Hospital of Chongqing Medical University between April 2018 and April 2025. A total of 234 patients were included in the study, comprising 65 patients with PA and 169 patients with pituitary adenomas.

**Figure 1:**
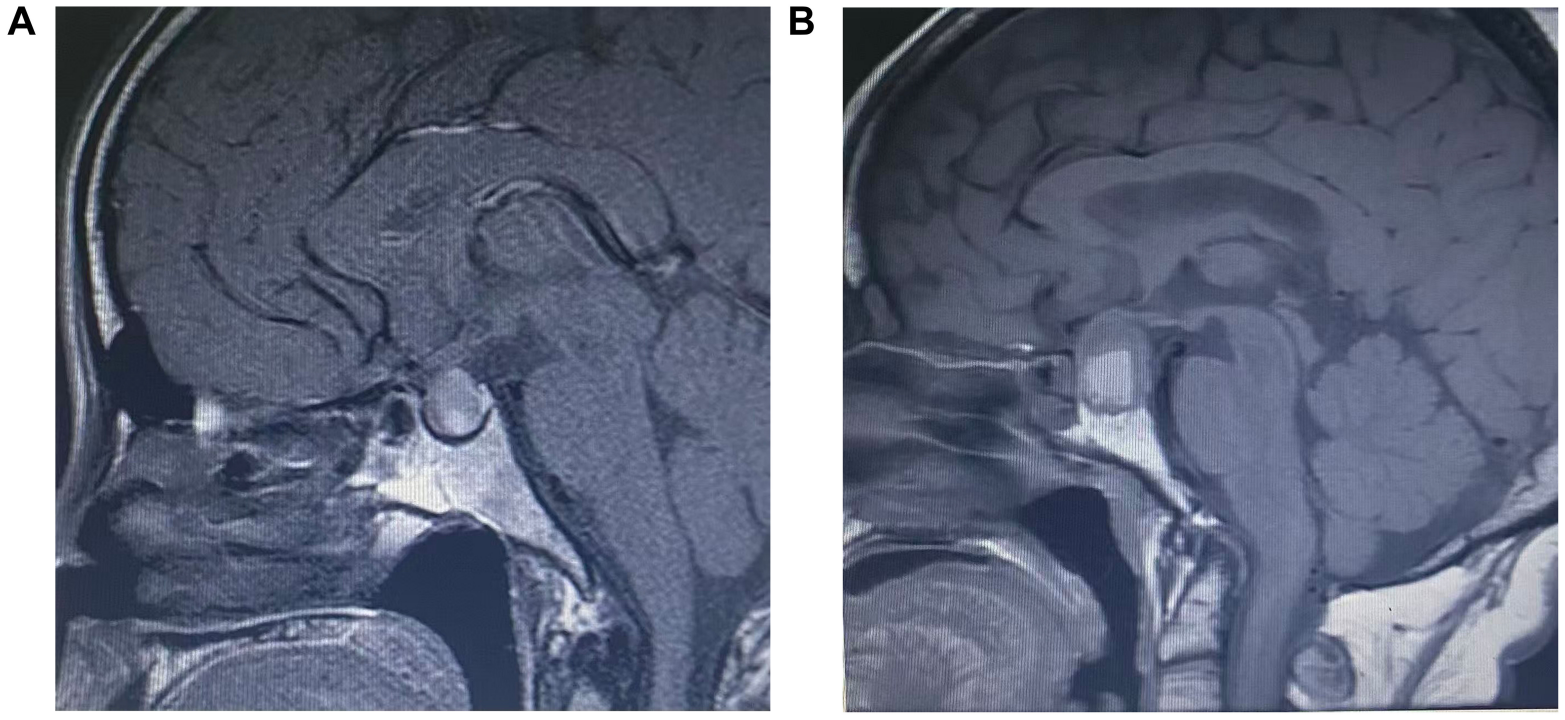
Acute pituitary apoplexy **(A)** and subclinical pituitary apoplexy **(B)** The images show the presentation of acute pituitary apoplexy **(A)** and subclinical pituitary apoplexy **(B)** on unenhanced T1-weighted images (T1WI), where the intrasellar mass shows hyperintensity, forming a signal level related to acute/subacute bleeding. T1WI: T1-weighted imaging.

PA cases were further classified into acute PA and subclinical PA based on clinical presentation (13). PA cases were further classified into acute PA and subclinical PA based on clinical presentation (13). Acute PA was defined as cases in which patients presented with acute symptoms related to PA, such as headache, visual impairment, and/or altered consciousness, and in whom PA was confirmed by imaging examinations (Figure 1A). Subclinical PA referred to cases without overt clinical symptoms, in which radiological evidence of hemorrhage and/or necrosis within the pituitary gland was incidentally detected on imaging studies (Figure 1B).

Inclusion Criteria: (1) Newly diagnosed patients; (2) Patients diagnosed with PA, defined as meeting at least two of the following three criteria: (1) Sudden onset of symptoms suggestive of PA, such as severe headache, vomiting, or visual impairment; (2)Radiological evidence of intrapituitary hemorrhage on imaging (MRI or CT; (3) Pathological confirmation of hemorrhage (5, 14); (3) Pituitary adenoma patients were included if imaging (MRI or CT) confirmed the presence of a pituitary lesion and postoperative pathology confirmed pituitary adenoma; (4) Patients who received treatment within 72 h of symptom onset and had initial blood samples collected within 24 h post-hemorrhage.

Exclusion Criteria: (1) Patients with incomplete clinical data; (2) Patients with the following comorbiditie: (1) A history of severe systemic diseases, such as cardiac, pulmonary, hepatic, or renal failure; (2) Presence of other intracranial or sellar region tumors, including craniopharyngioma and parasellar meningioma. This study was approved by the Ethics Committee of the Second Affiliated Hospital of Chongqing Medical University (approval date: April 14, 2025; approval number: 2025IIT188).

#### 2.2.2 Data collection

Clinical variables were extracted from the medical records database, including gender, age, hypertension, diabetes mellitus, smoking status, alcohol consumption, history of orthopedic or cardiac surgery, cardiovascular disease (CVD), anticoagulation therapy, dopamine agonist use, hemoglobin, platelet count, Knosp grade, tumor size, tumor type, activated partial thromboplastin time (APTT), prothrombin time (PT), fibrinogen, insulin-like growth factor 1 (IGF-1), growth hormone (GH), serum cortisol levels at 08:00, 16:00, and 00:00, adrenocorticotropic hormone (ACTH), luteinizing hormone (LH), follicle-stimulating hormone (FSH), prolactin (PRL), thyroid-stimulating hormone (TSH), and thyroid hormones.

For patients with PA, clinical manifestations at onset were recorded to distinguish acute PA from subclinical PA, and relevant clinical and laboratory parameters were compared between the two groups.

Hypertension was defined as the use of antihypertensive medications or a systolic blood pressure ≥140 mmHg or diastolic blood pressure ≥90 mmHg (15). Diabetes mellitus (DM) was diagnosed according to the 1999 WHO diagnostic criteria. All patients underwent computed tomography (CT) and/or magnetic resonance imaging (MRI). Three-dimensional MRI was used to assess tumor size and characteristics. Pituitary adenomas with a diameter ≥10 mm were classified as macroadenomas, whereas those < 10 mm were considered microadenomas (16). Tumor type was determined by the Department of Pathology of the Second Affiliated Hospital of Chongqing Medical University using standard immunohistochemical staining methods. Tumors were classified according to their immunohistochemical hormone expression profiles, including prolactin (PRL), growth hormone (GH), adrenocorticotropic hormone (ACTH), thyroid-stimulating hormone (TSH), follicle-stimulating hormone (FSH), and luteinizing hormone (LH). Serum PRL levels were measured using standard laboratory assays and categorized as low, normal, or elevated according to the reference ranges of the institutional laboratory. Specifically, normal PRL levels were defined as 5–25 ng/ml for non-pregnant women and 5–20 ng/ml for men. Functional pituitary adenomas were defined as tumors associated with clinical and biochemical evidence of hormone hypersecretion, supported by positive immunohistochemical staining for the corresponding hormone. Non-functioning pituitary adenomas were defined as tumors without clinical evidence of hormone hypersecretion, including immunohistochemically negative adenomas.

#### 2.2.3 Statistics

The study data were randomly split into a training set and a validation set at a ratio of 7:3 using the R package caret. The training set was used to screen variables and develop the nomogram model, while the validation set was used to assess the model's performance and stability. Categorical variables were presented as numbers and percentages, and differences between groups were compared using the Student's *t*-test, Mann-Whitney U test, or Chi-square test, depending on the type of variable. In the training set, univariate logistic regression analysis was used to identify variables significantly associated with PA (*P* < 0.05). These variables were then included in multivariate logistic regression analysis. A backward stepwise regression method was applied to retain the significant variables (*P* < 0.05) for the nomogram model.

In the nomogram, each variable corresponds to a score (typically ranging from 0 to 100) shown at the top of the dotted line. The total score for each patient is the sum of the scores of all variables (17). The receiver operating characteristic (ROC) curve was plotted based on the total score to determine the optimal cut-off value for predicting PA and to assess the model's discriminatory ability. ROC curve analysis was conducted in both the training set and the validation set to evaluate the model's discriminative performance (18). Additionally, the Hosmer-Lemeshow test was used to assess the model's calibration performance. A *P*-value > 0.05 indicates good calibration. The decision curve analysis (DCA) was performed to evaluate the clinical utility of the model at different threshold probabilities and to quantify the net benefit. In addition, potential differences in risk factors between acute PA and subclinical PA were explored.

Data management and statistical analysis were performed using R version 4.4.0. All statistical tests were two-sided, and a *P*-value < 0.05 was considered statistically significant.

## 3 Results

### 3.1 Meta-analysis

#### 3.1.1 Literature search

**Figure 2:**
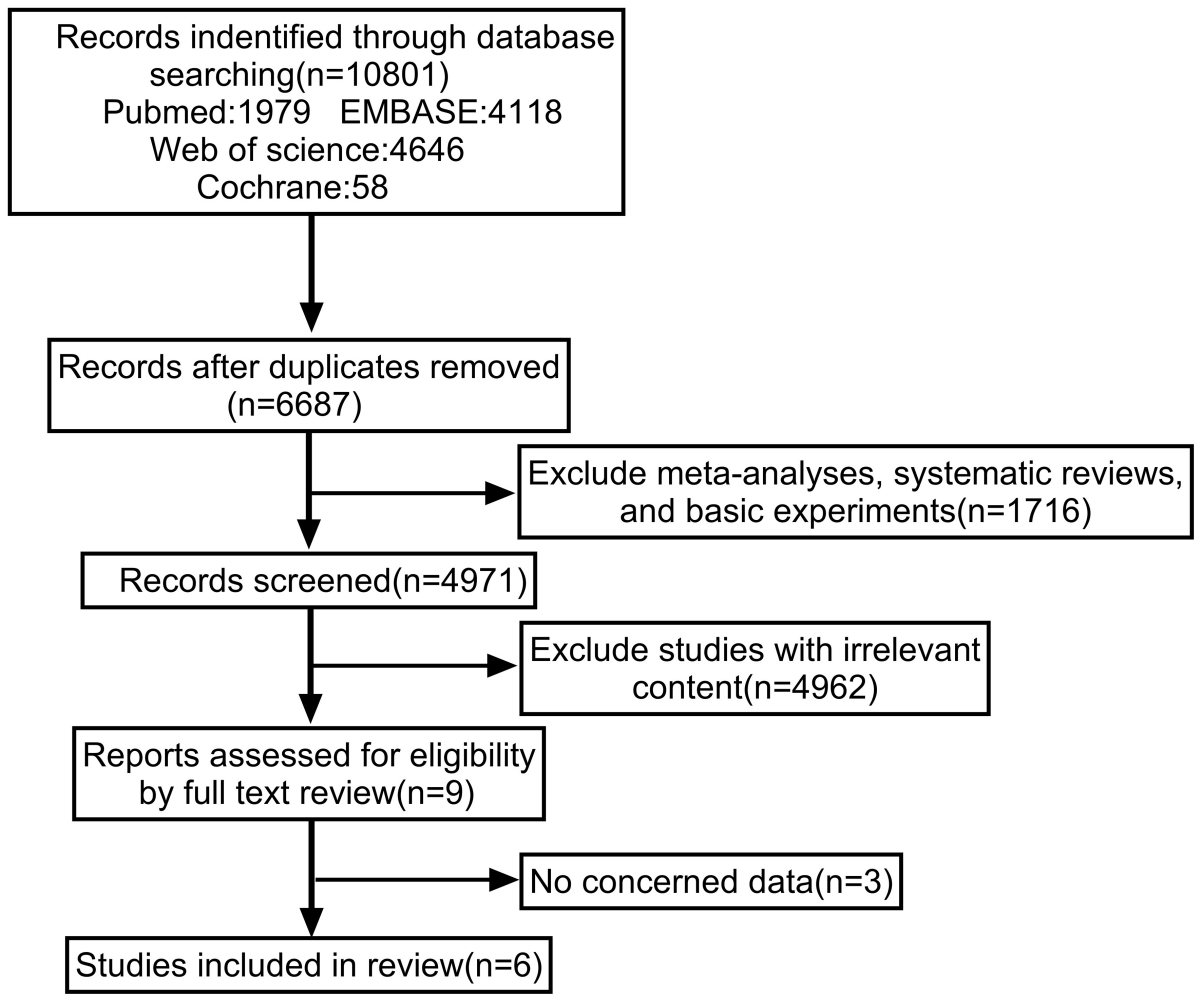
Literature screening flowchart.

The entire literature search process is shown in Figure 2. After searching PubMed, EMBASE, Web of Science, and Cochrane databases, a total of 10,801 articles were identified. After removing duplicates, 6,687 articles remained. Following the exclusion of meta-analyses, reviews, basic studies, and irrelevant titles and abstracts, 9 articles were selected for full-text review. Three articles were excluded due to lack of relevant data. Therefore, a total of 6 articles were ultimately included in our study, all of which were case-control studies.

#### 3.1.2 Study characteristics

In this meta-analysis, six studies published between 2012 and 2023 were included. These studies were conducted by reputable medical research institutions from three countries (four from China, one from Spain, and one from the United States), and all were retrospective case-control studies. A total of 1,352 patients with pituitary adenomas and 496 patients with PA were analyzed. The quality of all six studies was high, with Newcastle-Ottawa Scale (NOS) scores of 6 or above. Table 1 presents the relevant information for the included studies.

**Table 1:** Summary of characteristics of included studies.

**Author**	**Year**	**Area**	**Study design**	**Sample size (cases)**		**Exposure factors**	**NOS score**
				**Test group**	**Control group**		
(5)	2015	China	Retrospective	97	194	1.2.3	8
(8)	2023	Spain	Retrospective	60	185	1	7
(13)	2020	China	Retrospective	121	242	2.3.4	8
(20)	2023	America	Retrospective	77	265	1.5.7.10	7
(26)	2021	China	Retrospective	60	259	4.8.9	7
(27)	2012	China	Retrospective	81	207	2.3.6.7.11.12	7

(1)male, (2)non-functioning tumor, (3)macroadenoma, (4)Hypertension, (5)INR, (6) Malignant disease, (7)Dopamineagonist, (8)Recurrent pituitary adenoma, (9)Tumor diameter, (10)PTT, (11)Anticoagulant therapy, (12)End-stage renal disease.

#### 3.1.3 Data analysis

**Figure 3:**
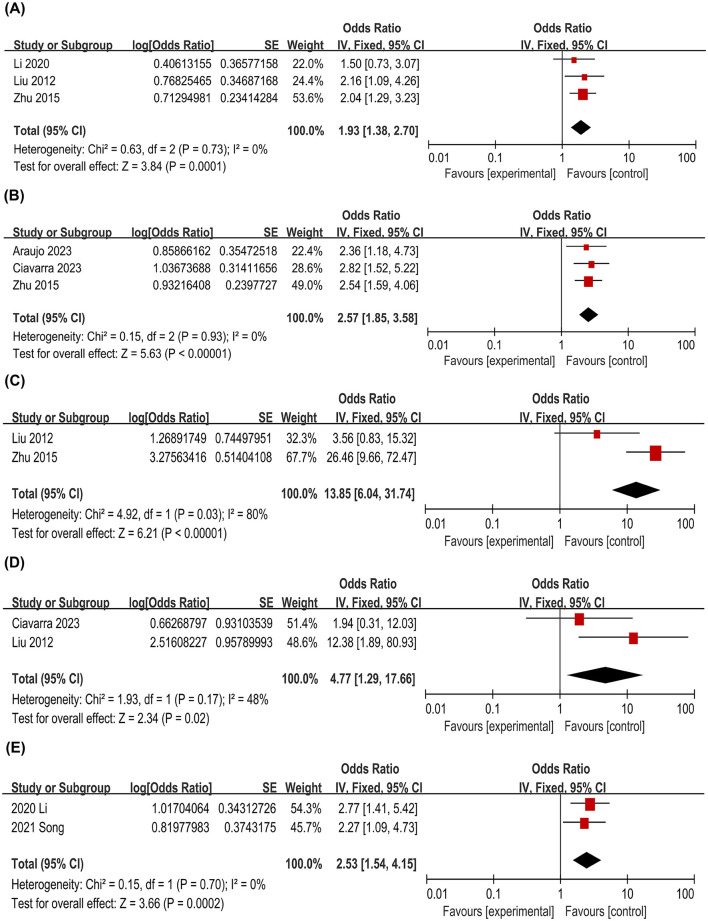
Meta-analysis of non-functioning pituitary adenomas and pituitary apoplexy **(A)**; Meta-analysis of male sex and pituitary apoplexy **(B)**; Meta-analysis of tumor size and pituitary apoplexy **(C)**; Meta-analysis of dopamine agonist therapy and pituitary apoplexy **(D)**; Meta-analysis of hypertension and pituitary apoplexy **(E)**.

Three studies evaluated the association between non-functioning pituitary adenomas and male sex with PA. Fixed-effect meta-analysis demonstrated significant associations for both non-functioning adenomas (OR = 1.93; 95% CI: 1.38–2.70; *P* = 0.0001) and male sex (OR = 2.57; 95% CI: 1.85–3.58; *P < 0.00001*) (Figures 3A, B). Additionally, two studies indicated a significant correlation between hypertension and PA (OR = 2.53; 95% CI: 1.54–4.15; *P* = 0.0002) (Figure 3E). In contrast, no significant associations were observed for dopamine agonist therapy and tumor size (Figures 3C, D).

#### 3.1.4 Publication bias

Furthermore, an assessment of publication bias was conducted, and no significant evidence of publication bias was observed.

### 3.2 Nomogram model

#### 3.2.1 Baseline characteristics of the study population

A total of 234 patients with pituitary adenomas were included in the retrospective cohort. Of these, 65 patients (27.78%) had a diagnosis of PA. Patients were randomly assigned to the training cohort (*n* = 163) and validation cohort (*n* = 71). Baseline demographic and clinical characteristics were comparable between the two cohorts (Table 2).

**Table 2:** Baseline characteristics of the training and validation sets.

**Variables**	**Total (*n* = 234)**	**test (*n* = 71)**	**train (*n* = 163)**	**Statistic**	***P***
Age, Mean ± SD	54.27 ± 13.55	56.24 ± 14.07	53.41 ± 13.28	*t* = 1.47	0.143
**Gender**, ***n*** **(%)**					
Female	116 (49.57)	30 (42.25)	86 (52.76)	χ^2^ = 2.18	0.139
Male	118 (50.43)	41 (57.75)	77 (47.24)		
**Hypertension**, ***n*** **(%)**					
No	174 (74.36)	47 (66.20)	127 (77.91)	χ^2^ = 3.56	0.059
Yes	60 (25.64)	24 (33.80)	36 (22.09)		
**Diabetes**, ***n*** **(%)**					
No	213 (91.03)	65 (91.55)	148 (90.80)	χ^2^ = 0.03	0.853
Yes	21 (8.97)	6 (8.45)	15 (9.20)		
**Smoke**, ***n*** **(%)**					
No	183 (78.21)	53 (74.65)	130 (79.75)	χ^2^ = 0.76	0.384
Yes	51 (21.79)	18 (25.35)	33 (20.25)		
**Drink wine, n (%)**					
No	215 (91.88)	62 (87.32)	153 (93.87)	χ^2^ = 2.84	0.092
Yes	19 (8.12)	9 (12.68)	10 (6.13)		
**History of orthopedic or cardiac surgery**, ***n*** **(%)**					
No	225 (96.15)	67 (94.37)	158 (96.93)	χ^2^ = 0.32	0.570
Yes	9 (3.85)	4 (5.63)	5 (3.07)		
**CVD**, ***n*** **(%)**					
No	228 (97.44)	69 (97.18)	159 (97.55)	χ^2^ = 0.00	1.000
Yes	6 (2.56)	2 (2.82)	4 (2.45)		
**Anticoagulation**, ***n*** **(%)**					
No	195 (83.33)	59 (83.10)	136 (83.44)	χ^2^ = 0.00	0.949
Yes	39 (16.67)	12 (16.90)	27 (16.56)		
**Dopamine agonist**, ***n*** **(%)**					
No	223 (95.30)	70 (98.59)	153 (93.87)	χ^2^ = 1.52	0.217
Yes	11 (4.70)	1 (1.41)	10 (6.13)		
**Hemoglobin**, ***n*** **(%)**					
Normal	190 (81.20)	58 (81.69)	132 (80.98)	χ^2^ = 0.02	0.899
Low	44 (18.80)	13 (18.31)	31 (19.02)		
**Platelet**, ***n*** **(%)**					
Normal	227 (97.01)	69 (97.18)	158 (96.93)	χ^2^ = 0.00	1.000
Low	7 (2.99)	2 (2.82)	5 (3.07)		
**Knosp grade**, ***n*** **(%)**					
0-2	136 (58.12)	36 (50.70)	100 (61.35)	χ^2^ = 2.30	0.129
3-4	98 (41.88)	35 (49.30)	63 (38.65)		
**Size**, ***n*** **(%)**					
Microadenoma	39 (16.67)	11 (15.49)	28 (17.18)	χ^2^ = 0.10	0.751
Macroadenoma	195 (83.33)	60 (84.51)	135 (82.82)		
**Type**, ***n*** **(%)**					
Non-functional adenoma	102 (43.59)	37 (52.11)	65 (39.88)	χ^2^ = 3.01	0.083
Functional adenoma	132 (56.41)	34 (47.89)	98 (60.12)		
**APTT**, ***n*** **(%)**					
Normal	225 (96.15)	69 (97.18)	156 (95.71)	χ^2^ = 0.03	0.865
Abnormal	9 (3.85)	2 (2.82)	7 (4.29)		
**PT**, ***n*** **(%)**					
Normal	223 (95.30)	68 (95.77)	155 (95.09)	χ^2^ = 0.00	1.000
Abnormal	11 (4.70)	3 (4.23)	8 (4.91)		
**Fibrinogen**, ***n*** **(%)**					
Normal	219 (93.59)	66 (92.96)	153 (93.87)	χ^2^ = 0.00	1.000
Abnormal	15 (6.41)	5 (7.04)	10 (6.13)		
**IGF1**, ***n*** **(%)**					
Normal	187 (79.91)	59 (83.10)	128 (78.53)	χ^2^ = 0.65	0.724
Low	31 (13.25)	8 (11.27)	23 (14.11)		
High	16 (6.84)	4 (5.63)	12 (7.36)		
**GH**, ***n*** **(%)**					
Normal	192 (82.05)	58 (81.69)	134 (82.21)	χ^2^ = 0.44	0.802
Low	26 (11.11)	9 (12.68)	17 (10.43)		
High	16 (6.84)	4 (5.63)	12 (7.36)		
**Cortisol 8a.m.**, ***n*** **(%)**					
Normal	162 (69.23)	43 (60.56)	119 (73.01)	χ^2^ = 4.03	0.133
Low	51 (21.79)	21 (29.58)	30 (18.40)		
High	21 (8.97)	7 (9.86)	14 (8.59)		
**Cortisol 16pm**, ***n*** **(%)**					
Normal	147 (62.82)	41 (57.75)	106 (65.03)	χ^2^ = 3.25	0.197
Low	52 (22.22)	21 (29.58)	31 (19.02)		
High	35 (14.96)	9 (12.68)	26 (15.95)		
**Cortisol 0a.m.**, ***n*** **(%)**					
Normal	229 (97.86)	69 (97.18)	160 (98.16)	–	0.713
Low	4 (1.71)	2 (2.82)	2 (1.23)		
High	1 (0.43)	0 (0.00)	1 (0.61)		
**ACTH**, ***n*** **(%)**					
Normal	208 (88.89)	64 (90.14)	144 (88.34)	–	1.000
High	15 (6.41)	4 (5.63)	11 (6.75)		
Low	11 (4.70)	3 (4.23)	8 (4.91)		
**LH**, ***n*** **(%)**					
Normal	100 (42.74)	31 (43.66)	69 (42.33)	χ^2^ = 0.54	0.763
Low	121 (51.71)	35 (49.30)	86 (52.76)		
High	13 (5.56)	5 (7.04)	8 (4.91)		
**FSH**, ***n*** **(%)**					
Normal	190 (81.20)	58 (81.69)	132 (80.98)	–	1.000
High	39 (16.67)	12 (16.90)	27 (16.56)		
Low	5 (2.14)	1 (1.41)	4 (2.45)		
**PRL**, ***n*** **(%)**					
Normal	137 (58.55)	39 (54.93)	98 (60.12)	χ^2^ = 1.33	0.514
**TSH**, ***n*** **(%)**					
Normal	206 (88.03)	62 (87.32)	144 (88.34)	–	0.306
Low	13 (5.56)	6 (8.45)	7 (4.29)		
High	15 (6.41)	3 (4.23)	12 (7.36)		
**Thyroid hormones**, ***n*** **(%)**					
Normal	211 (90.17)	65 (91.55)	146 (89.57)	–	0.579
Low	21 (8.97)	5 (7.04)	16 (9.82)		
High	2 (0.85)	1 (1.41)	1 (0.61)		

CVD, cardiovascular disease; APTT, activated partial thromboplastin time; PT, prothrombin time; IGF1, Insulin-like growth factor-1; GH, growth hormone; ACTH, adrenocorticotropic hormone; LH, luteinizing hormone; FSH, follicle-stimulating hormone; PRL, prolactin; TSH), thyroid-stimulating hormone.

#### 3.2.2 Selection of predictive factors in the nomogram

Univariate analysis showed that, in the training cohort, male sex, hypertension, smoking, anticoagulant therapy, functional pituitary adenomas, abnormal APTT, reduced cortisol at 16:00, and elevated PRL were significantly associated with the occurrence of PA. Based on the final model selected using the lowest Akaike information criterion (AIC), gender (OR = 5.49, 95% CI: 1.99–15.12), hypertension (OR = 4.79, 95% CI: 1.42–16.14), anticoagulant therapy (OR = 6.17, 95% CI: 1.63–23.32), tumor type (OR = 0.08, 95% CI: 0.03–0.23), and PRL (OR = 0.19, 95% CI: 0.05–0.73) were identified as independent factors associated with PA (Table 3).

**Table 3:** Univariate and multivariate logistic regression analysis of risk factors for pituitary apoplexy.

**Variables**	**Univariable logistic regression**					**Multivariable logistic regression**				
	**β**	**S.E**	**Z**	**P**	**CI**	**β**	**S.E**	**Z**	**P**	**CI**
Age, Mean ± SD	0.02	0.01	1.13	0.257	1.02 (0.99–1.05)					
**Gender**, ***n*** **(%)**										
Female					1.00 (Reference)					1.00 (Reference)
Male	1.15	0.39	2.91	0.004	3.14 (1.45–6.80)	1.70	0.52	3.30	< .001	5.49 (1.99–15.12)
**Hypertension**, ***n*** **(%)**										
No					1.00 (Reference)					1.00 (Reference)
Yes	0.84	0.41	2.03	0.043	2.31 (1.03–5.18)	1.57	0.62	2.52	0.012	4.79 (1.42–16.14)
**Diabetes**, ***n*** **(%)**										
No					1.00 (Reference)					
Yes	0.20	0.62	0.32	0.747	1.22 (0.36–4.08)					
**Smoke**, ***n*** **(%)**										
No					1.00 (Reference)					
Yes	1.18	0.42	2.82	0.005	3.25 (1.43–7.39)					
**Drink wine**, ***n*** **(%)**										
No					1.00 (Reference)					
Yes	0.85	0.67	1.26	0.209	2.33 (0.62–8.75)					
**History of orthopedic or cardiac surgery**, ***n*** **(%)**										
No					1.00 (Reference)					
Yes	−0.20	1.13	−0.18	0.859	0.82 (0.09–7.54)					
**CVD**, ***n*** **(%)**										
No					1.00 (Reference)					
Yes	1.23	1.02	1.21	0.227	3.42 (0.46–25.12)					
**Anticoagulation**, ***n*** **(%)**										
No					1.00 (Reference)					1.00 (Reference)
Yes	1.02	0.45	2.28	0.022	2.78 (1.16–6.66)	1.82	0.68	2.68	0.007	6.17 (1.63–23.32)
**Dopamine agonist**, ***n*** **(%)**										
No					1.00 (Reference)					
Yes	−0.21	0.81	−0.26	0.798	0.81 (0.17–4.00)					
**Hemoglobin**, ***n*** **(%)**										
Normal					1.00 (Reference)					
Low	−0.55	0.53	−1.04	0.298	0.58 (0.20–1.62)					
**Platelet**, ***n*** **(%)**										
Normal					1.00 (Reference)					
Low	−0.20	1.13	−0.18	0.859	0.82 (0.09–7.54)					
**Knosp grade**, ***n*** **(%)**										
0–2					1.00 (Reference)					
3–4	0.19	0.38	0.50	0.618	1.21 (0.58–2.53)					
**Size**, ***n*** **(%)**										
Microadenoma					1.00 (Reference)					
Macroadenoma	1.07	0.64	1.67	0.095	2.92 (0.83–10.26)					
**Type**, ***n*** **(%)**										
Non-functional adenoma					1.00 (Reference)					1.00 (Reference)
Functional adenoma	−2.27	0.44	−5.09	< .001	0.10 (0.04–0.25)	−2.55	0.56	−4.56	< .001	0.08 (0.03–0.23)
**APTT**, ***n*** **(%)**										
Normal					1.00 (Reference)					
Abnormal	2.23	0.86	2.60	0.009	9.32 (1.73–50.21)					
**PT**, ***n*** **(%)**										
Normal					1.00 (Reference)					
Abnormal	0.72	0.76	0.96	0.339	2.06 (0.47–9.04)					
**Fibrinogen**, ***n*** **(%)**										
Normal					1.00 (Reference)					
Abnormal	1.29	0.66	1.95	0.051	3.64 (0.99–13.32)					
**IGF1**, ***n*** **(%)**										
Normal					1.00 (Reference)					
Low	−0.18	0.55	−0.33	0.738	0.83 (0.29–2.43)					
High	−1.30	1.06	−1.22	0.222	0.27 (0.03–2.19)					
**GH**, ***n*** **(%)**										
Normal					1.00 (Reference)					
Low	−0.06	0.61	−0.10	0.921	0.94 (0.29–3.09)					
High	−1.28	1.06	−1.20	0.229	0.28 (0.03–2.24)					
**Cortisol 8a.m.**, ***n*** **(%)**										
Normal					1.00 (Reference)					
Low	0.83	0.44	1.87	0.061	2.29 (0.96–5.45)					
High	0.08	0.69	0.11	0.912	1.08 (0.28–4.18)					
**Cortisol 16pm**, ***n*** **(%)**										
Normal					1.00 (Reference)					
Low	1.20	0.44	2.70	0.007	3.31 (1.39–7.89)					
High	0.32	0.53	0.60	0.549	1.37 (0.49–3.88)					
**Cortisol 0a.m.**, ***n*** **(%)**										
Normal					1.00 (Reference)					
Low	1.20	1.43	0.84	0.400	3.32 (0.20–54.45)					
High	−13.36	882.74	−0.02	0.988	0.00 (0.00–Inf)					
**ACTH**, ***n*** **(%)**										
Normal					1.00 (Reference)					
High	0.73	0.66	1.11	0.265	2.08 (0.57–7.58)					
Low	0.78	0.76	1.03	0.302	2.19 (0.50–9.66)					
**LH**, ***n*** **(%)**										
Normal					1.00 (Reference)					
Low	−0.29	0.37	−0.78	0.434	0.75 (0.36–1.55)					
High	−16.60	1398.72	−0.01	0.991	0.00 (0.00–Inf)					
**FSH**, ***n*** **(%)**										
Normal					1.00 (Reference)					
High	−0.65	0.58	−1.13	0.260	0.52 (0.17–1.62)					
Low	0.00	1.17	0.00	1.000	1.00 (0.10–9.95)					
**PRL**, ***n*** **(%)**										
Normal					1.00 (Reference)					1.00 (Reference)
Low	−1.48	0.57	−2.60	0.009	0.23 (0.08–0.70)	−1.67	0.69	−2.43	0.015	0.19 (0.05–0.73)
High	0.83	0.56	1.48	0.140	2.30 (0.76–6.96)	0.50	0.70	0.72	0.469	1.66 (0.42–6.51)
**TSH**, ***n*** **(%)**										
Normal					1.00 (Reference)					
Low	0.89	0.79	1.12	0.261	2.43 (0.52–11.38)					
High	−1.22	1.06	−1.15	0.249	0.29 (0.04–2.36)					
**Thyroid hormones**, ***n*** **(%)**										
Normal					1.00 (Reference)					
Low	−0.31	0.67	−0.47	0.641	0.73 (0.20–2.72)					
High	−13.41	882.74	−0.02	0.988	0.00 (0.00–Inf)					

CVD, cardiovascular disease; APTT, activated partial thromboplastin time; PT, prothrombin time; IGF1, Insulin-like growth factor-1; GH, growth hormone; ACTH, adrenocorticotropic hormone; LH, luteinizing hormone; FSH, follicle-stimulating hormone; PRL, prolactin; TSH), thyroid-stimulating hormone.

#### 3.2.3 Development and validation of the nomogram model

**Figure 4:**
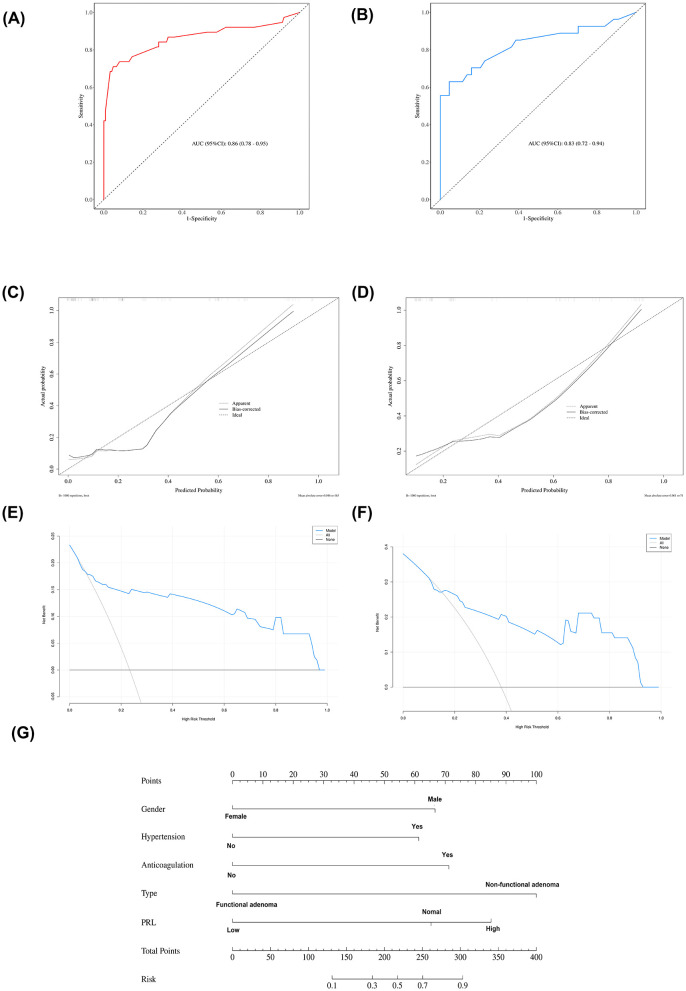
Performance of the predictive model for pituitary apoplexy in patients with pituitary adenomas. The ROC curves demonstrate that the area under the curve (AUC) was 0.86 (95% CI: 0.78–0.95) in the training cohort **(A)** and 0.83 (95% CI: 0.72–0.94) in the validation cohort **(B)**. The calibration curves of the nomogram showed good agreement between predicted and observed outcomes in both the training cohort **(C)** and the validation cohort **(D)**. The y-axis represents the net benefit. The blue line represents the nomogram. The thin solid line represents the assumption that all patients have pituitary apoplexy. The thick solid line represents the assumption that no patients have pituitary apoplexy **(E, F)**. To use the nomogram, draw a vertical line from each variable to the “Points” axis to determine its score. Sum the scores and locate the total on the “Total Points” axis. Finally, draw a vertical line down to the “Diagnostic Possibility” axis to estimate the predicted probability of pituitary apoplexy in patients with pituitary adenomas **(G)**.For example, a male (66.5 points) with a history of hypertension (61 points), no anticoagulation therapy (0 points), post-operative diagnosis of a functional adenoma (0 points), and elevated prolactin levels (85 points) has a total of 212.5 points. Based on the nomogram, the predicted probability of pituitary apoplexy for this patient is 47%.*prolactin (PRL).

Based on the results of multivariable logistic regression analysis, a nomogram model was developed to predict the probability of PA in patients (Figure 4G). By summing the scores assigned to each predictive variable, the individual risk of PA could be calculated. To evaluate the discriminative ability of the nomogram, receiver operating characteristic (ROC) curves were constructed. The area under the curve (AUC) was 0.86 (95% CI: 0.78–0.95) in the training cohort (Figure 4A) and 0.83 (95% CI: 0.72–0.94) in the validation cohort (Figure 4B). The optimal cutoff value of the model was determined according to the Youden index and was set at 0.66. In the training cohort, the sensitivity and specificity were 0.71 and 0.95, respectively, while in the validation cohort they were 0.67 and 0.86, respectively.

The calibration curves of the nomogram demonstrated good agreement between the predicted and observed probabilities (Figures 4C, D). The Hosmer–Lemeshow goodness-of-fit test yielded *P* values of 1 and 0.272 in the training and validation cohorts, respectively, indicating a good model fit with no significant deviation. In addition, to assess the clinical feasibility of the model, we performed decision curve analysis (DCA) (Figures 4E, F). Specifically, in the training cohort (Figure 4E), when the threshold probability ranges from 7% to 96%, using the nomogram developed in this study to predict PA provides greater clinical benefit.

#### 3.2.4 Comparison between acute and subclinical PA

The clinical characteristics of patients with acute PA and subclinical PA are summarized in Table 4. Overall, most patients were diagnosed with acute PA, accounting for 48 cases (73.85%), while 17 patients (26.15%) had subclinical PA. Except for a history of anticoagulant therapy, Knosp grade, and tumor size, no statistically significant differences were observed between patients with acute PA and those with subclinical PA in other clinical or laboratory parameters.

**Table 4:** Comparison between acute and subclinical pituitary apoplexy.

**Variables**	**Total (*n* = 65)**	**Subclinical apoplexy (*n* = 17)**	**Acute apoplexy (*n* = 48)**	**Statistic**	***P***
Age, Mean ± SD	56.37 ± 14.44	53.41 ± 16.04	57.42 ± 13.86	*t* = −0.98	0.330
**Gender**, ***n*** **(%)**					
Female	21 (32.31)	5 (29.41)	16 (33.33)	χ^2^ = 0.09	0.766
Male	44 (67.69)	12 (70.59)	32 (66.67)		
**Hypertension**, ***n*** **(%)**					
No	39 (60.00)	11 (64.71)	28 (58.33)	χ^2^ = 0.21	0.645
Yes	26 (40.00)	6 (35.29)	20 (41.67)		
**Diabetes**, ***n*** **(%)**					
No	59 (90.77)	16 (94.12)	43 (89.58)	χ^2^ = 0.00	0.946
Yes	6 (9.23)	1 (5.88)	5 (10.42)		
**Smoke**, ***n*** **(%)**					
No	44 (67.69)	13 (76.47)	31 (64.58)	χ^2^ = 0.81	0.368
Yes	21 (32.31)	4 (23.53)	17 (35.42)		
**Drink wine**, ***n*** **(%)**					
No	57 (87.69)	15 (88.24)	42 (87.50)	χ^2^ = 0.00	1.000
Yes	8 (12.31)	2 (11.76)	6 (12.50)		
**History of orthopedic or cardiac surgery**, ***n*** **(%)**					
No	62 (95.38)	17 (100.00)	45 (93.75)	–	0.561
Yes	3 (4.62)	0 (0.00)	3 (6.25)		
**CVD**, ***n*** **(%)**					
No	61 (93.85)	17 (100.00)	44 (91.67)	χ^2^ = 0.41	0.521
Yes	4 (6.15)	0 (0.00)	4 (8.33)		
**Anticoagulation**, ***n*** **(%)**					
No	45 (69.23)	16 (94.12)	29 (60.42)	χ^2^ = 6.69	0.010
Yes	20 (30.77)	1 (5.88)	19 (39.58)		
**Dopamine agonist**, ***n*** **(%)**					
No	63 (96.92)	17 (100.00)	46 (95.83)	–	1.000
Yes	2 (3.08)	0 (0.00)	2 (4.17)		
**Hemoglobin**, ***n*** **(%)**					
Normal	54 (83.08)	16 (94.12)	38 (79.17)	χ^2^ = 1.07	0.300
Low	11 (16.92)	1 (5.88)	10 (20.83)		
**Platelet**, ***n*** **(%)**					
Normal	63 (96.92)	16 (94.12)	47 (97.92)	–	0.458
Low	2 (3.08)	1 (5.88)	1 (2.08)		
**Knosp grade**, ***n*** **(%)**					
0–2	35 (53.85)	13 (76.47)	22 (45.83)	χ^2^ = 4.74	0.029
3–4	30 (46.15)	4 (23.53)	26 (54.17)		
**Size**, ***n*** **(%)**					
Microadenoma	6 (9.23)	5 (29.41)	1 (2.08)	χ^2^ = 8.17	0.004
Macroadenoma	59 (90.77)	12 (70.59)	47 (97.92)		
**Type**, ***n*** **(%)**					
Non-functional adenoma	51 (78.46)	13 (76.47)	38 (79.17)	χ^2^ = 0.00	1.000
**APTT**, ***n*** **(%)**					
Normal	58 (89.23)	16 (94.12)	42 (87.50)	χ^2^ = 0.09	0.763
Abnormal	7 (10.77)	1 (5.88)	6 (12.50)		
**PT**, ***n*** **(%)**					
Normal	60 (92.31)	15 (88.24)	45 (93.75)	χ^2^ = 0.04	0.839
Abnormal	5 (7.69)	2 (11.76)	3 (6.25)		
**Fibrinogen**, ***n*** **(%)**					
Normal	56 (86.15)	17 (100.00)	39 (81.25)	χ^2^ = 2.29	0.130
Abnormal	9 (13.85)	0 (0.00)	9 (18.75)		
**IGF1**, ***n*** **(%)**					
Normal	53 (81.54)	14 (82.35)	39 (81.25)	–	0.627
Low	9 (13.85)	3 (17.65)	6 (12.50)		
High	3 (4.62)	0 (0.00)	3 (6.25)		
**GH**, ***n*** **(%)**					
Normal	54 (83.08)	15 (88.24)	39 (81.25)	–	0.854
Low	8 (12.31)	2 (11.76)	6 (12.50)		
High	3 (4.62)	0 (0.00)	3 (6.25)		
**Cortisol 8a.m.**, ***n*** **(%)**					
Normal	40 (61.54)	11 (64.71)	29 (60.42)	–	0.330
Low	19 (29.23)	6 (35.29)	13 (27.08)		
High	6 (9.23)	0 (0.00)	6 (12.50)		
**Cortisol 16pm**, ***n*** **(%)**					
Normal	32 (49.23)	11 (64.71)	21 (43.75)	χ^2^ = 2.31	0.315
Low	24 (36.92)	4 (23.53)	20 (41.67)		
High	9 (13.85)	2 (11.76)	7 (14.58)		
**Cortisol 0a.m.**, ***n*** **(%)**					
Normal	63 (96.92)	16 (94.12)	47 (97.92)	–	0.458
Low	2 (3.08)	1 (5.88)	1 (2.08)		
**ACTH**, ***n*** **(%)**					
Normal	52 (80.00)	16 (94.12)	36 (75.00)	–	0.300
High	7 (10.77)	1 (5.88)	6 (12.50)		
Low	6 (9.23)	0 (0.00)	6 (12.50)		
**LH**, ***n*** **(%)**					
Normal	33 (50.77)	11 (64.71)	22 (45.83)	–	0.456
Low	31 (47.69)	6 (35.29)	25 (52.08)		
High	1 (1.54)	0 (0.00)	1 (2.08)		
**FSH**, ***n*** **(%)**					
Normal	57 (87.69)	16 (94.12)	41 (85.42)	–	0.214
High	6 (9.23)	0 (0.00)	6 (12.50)		
Low	2 (3.08)	1 (5.88)	1 (2.08)		
**PRL**, ***n*** **(%)**					
Normal	42 (64.62)	13 (76.47)	29 (60.42)	–	0.625
**TSH**, ***n*** **(%)**					
Normal	56 (86.15)	15 (88.24)	41 (85.42)	–	1.000
Low	8 (12.31)	2 (11.76)	6 (12.50)		
High	1 (1.54)	0 (0.00)	1 (2.08)		
**Thyroid hormones**, ***n*** **(%)**					
Normal	59 (90.77)	17 (100.00)	42 (87.50)	χ^2^ = 1.09	0.297
Low	6 (9.23)	0 (0.00)	6 (12.50)		

CVD, cardiovascular disease; APTT, activated partial thromboplastin time; PT, prothrombin time; IGF1, Insulin-like growth factor-1; GH, growth hormone; ACTH, adrenocorticotropic hormone; LH, luteinizing hormone; FSH, follicle-stimulating hormone; PRL, prolactin; TSH), thyroid-stimulating hormone.

## 4 Discussion

PA is an acute clinical syndrome with a multifactorial pathogenesis, commonly presenting with sudden-onset headache, visual impairment, ophthalmoplegia, and altered consciousness (1, 2). Although numerous studies have explored potential risk factors, their findings have been inconsistent (3). To address this gap, our study combined a meta-analysis and a retrospective cohort analysis to identify independent risk factors and construct a clinically useful predictive nomogram.

The pathogenesis of PA involves multiple factors, with some variability across different studies. Reported risk factors can be broadly categorized as follows: (1) Tumor-related factors: Larger tumor size, specific tumor types, and more invasive tumors have been associated with an increased risk of PA(4,5). (2) Hemodynamic changes: Conditions such as hypertension, diabetes, and increased intracranial pressure may disrupt pituitary blood supply, thereby elevating the risk of PA(6,7). (3) Endocrine stimulation: Pregnancy, childbirth, exogenous hormone therapy, and stimulation tests for hormone secretion have been implicated as potential triggers (19). (4) Anticoagulation and hematologic abnormalities: Anticoagulant therapy, antiplatelet medications, and coagulation disorders may contribute to an increased risk of intratumoral hemorrhage (13, 20, 21). These factors may act independently or interact synergistically, collectively influencing the risk of PA.

Our meta-analysis identified three significant risk factors for PA: non-functioning pituitary adenomas, male sex, and hypertension. These findings are consistent with prior studies, but provide stronger evidence through data synthesis and pooled effect sizes. The nomogram model developed from our institutional cohort further validated the predictive value of these factors and additionally identified a history of anticoagulant therapy and PRL as important predictors.

With the widespread application of imaging and pathological examinations, subclinical PA has received increasing attention and is often characterized by the absence of typical clinical manifestations such as severe headache, visual impairment, or neurological deficits (8, 13). These “silent” apoplexy events may share some risk factors with symptomatic PA, but the two entities are not completely overlapping, as symptomatic PA represents a true neuroendocrine emergency (1, 2). In our study, a history of anticoagulant therapy, Knosp grade, and tumor size differed significantly between acute and subclinical PA, suggesting that these factors may be associated with the clinical presentation and severity of PA. A recent systematic review by Kajal et al. also highlighted the spectrum of non–pregnancy-related risk factors for PA (22) and emphasized the need for future studies to separately evaluate risk profiles for symptomatic and asymptomatic presentations. Large-scale prospective studies are still warranted to determine whether the risk factors identified in the present study can be translated into reliable clinical predictors for symptomatic PA requiring urgent intervention (23).

Existing studies generally report a higher prevalence of PA in male patients compared to females (5, 13, 20, 24). For example, a study by Möller-Goede DL et al. (25) found that the incidence of PA in men (12%) was approximately three times higher than in women (4%). This disparity may be attributed to factors such as the higher prevalence of cardiovascular diseases in men, endothelial dysfunction, and the potential influence of androgens on the vascular system, all of which could increase the risk of hemorrhage or infarction.

In our nomogram model, tumor type emerged as a significant factor associated with PA. Interestingly, the role of tumor type has been debated in previous studies. Nonfunctioning pituitary adenomas (NFPAs) have traditionally been regarded as more prone to apoplexy. For instance, Möller-Goede et al. (25) reported that 76% of apoplexy cases occurred in patients with NFPAs. In contrast, Mou et al. found a higher proportion of functioning adenomas among apoplexy patients (26). In our study, consistent with the majority of previous evidence, NFPAs were identified as an independent risk factor for PA, with the meta-analysis indicating a 1.93-fold increased risk, and this association was further validated in our institutional cohort. The potential explanation may relate to the growth pattern and vascular supply of NFPAs. Unlike functioning adenomas, which are subject to endogenous hormonal regulation, NFPAs may exhibit more rapid tumor expansion and are more vulnerable to intratumoral hemorrhage or ischemic necrosis (27).

In contrast, the risk of apoplexy in patients with functional pituitary adenomas is lower, suggesting that clinical intervention with medication can help reduce apoplexy risk. Pharmacological treatments not only control hormone levels and reduce tumor volume but also improve vascular stability within the tumor, thereby partially preventing acute tumor expansion and apoplexy (28–31). These findings highlight the importance of early identification of functional adenomas and appropriate medical management, which not only allows precise hormonal control but may also clinically reduce the risk of PA.

This study found that elevated serum PRL levels were significantly associated with the occurrence of PA. Previous studies have shown that markedly increased PRL levels are often correlated with larger tumor volume, more pronounced mass effect, and a higher propensity for intratumoral hemorrhage, particularly in prolactin-secreting pituitary adenomas (32). In addition, PRL elevation resulting from pituitary stalk compression is widely regarded as an important surrogate marker of tumor burden and mass effect, both of which have been repeatedly demonstrated to play key roles in the pathogenesis of PA (16). From a pathophysiological perspective, tumors associated with elevated PRL levels are often characterized by relatively insufficient blood supply, increased intratumoral pressure, and fragile neovascular structures, rendering them more susceptible to ischemic or hemorrhagic events (1, 2, 33). Therefore, PRL elevation may represent not only an endocrine abnormality but also a potential biomarker for risk stratification and clinical management of PA. Nevertheless, its predictive value and underlying mechanisms require further confirmation through prospective studies.

Dopamine agonists (DAs) have been frequently reported in case studies and small series as potential triggers of PA (1). Clinical cases often attribute DA-associated apoplexy to rapid tumor shrinkage, lactotroph degeneration or necrosis, and subsequent intratumoral hemorrhage or infarction. Mechanistic studies suggest that DAs may induce adenoma cell apoptosis and vascular remodeling, resulting in localized fragile blood supply and triggering apoplexy (34). However, large-scale reviews and retrospective cohort studies indicate that routine DA therapy, particularly for prolactinomas, does not significantly increase apoplexy risk (22, 35). Consistently, our meta-analysis (21, 36) and nomogram did not demonstrate an association between DA therapy and apoplexy, highlighting the need for further mechanistic research and prospective data to clarify the role of DAs in the occurrence of PA.

Due to microvascular disease and abnormal blood supply changes (27), diabetes and hypertension are often considered major risk factors for vascular diseases, potentially increasing the risk of PA. Hypertension can disrupt the microvascular supply to the pituitary gland, making it more vulnerable to hemorrhage or infarction under stress (6, 7, 14). The meta-analysis and the nomogram in this study both demonstrated an association between hypertension and PA (14, 37). Our findings reinforce the importance of blood pressure control in patients with pituitary adenomas, especially those with large or invasive tumors.

Tumor size has long been recognized as a key factor in the risk of pituitary apoplexy. Larger tumors are more likely to exceed their vascular supply or compress surrounding structures, making them more prone to ischemia and hemorrhage (5, 14, 38–40). For instance, Li et al. (14) reported that macroadenomas with a diameter greater than 2 cm were associated with an increased risk of PA. Similarly, Zhu et al. (5) and Liu et al. (36) found that macroadenomas larger than 1 cm were linked to a significantly higher incidence of PA compared to microadenomas. Additionally, the Knosp grading system is used to assess the extent of pituitary adenoma invasion into the cavernous sinus, with higher grades indicating deeper invasion. Garcia-Feijoo et al. (23) identified a significant association between severe PA and a Knosp grade ≥2, suggesting that a higher Knosp grade may elevate the risk of severe PA.

Anticoagulant therapy remains a controversial risk factor for PA. A retrospective study including 342 patients with pituitary adenomas found that antiplatelet and anticoagulant therapy (AP/AC) did not significantly increase the incidence of apoplexy (21). However, in our nomogram model, anticoagulant therapy was identified as an independent predictor, and several case reports and retrospective studies have suggested that anticoagulant or antiplatelet therapy may trigger PA (41, 42). The potential mechanisms may include: the vascular structure within pituitary adenomas is fragile and prone to bleeding, and anticoagulants can impair coagulation, leading to rupture of fragile vessels and intratumoral hemorrhage or necrosis; in an anticoagulated state, microbleeds may be difficult to stop and may gradually expand to clinically apparent apoplexy; moreover, anticoagulant therapy is often used in patients with advanced age, hypertension, or cardiovascular disease, which may further increase bleeding risk through a cumulative effect of multiple risk factors (43). Although evidence remains limited and controversial, our findings suggest that anticoagulant/antiplatelet therapy should be used with caution in patients with pituitary adenomas, and close monitoring and careful risk–benefit assessment are warranted when such therapy is necessary.

During model development, we prioritized variables with clear clinical relevance and easy accessibility in routine practice, including sex, tumor type, tumor size, Knosp grade, history of hypertension, history of anticoagulant therapy, and serum PRL levels. All of these parameters are derived from standard radiological assessments, medical history collection, and basic laboratory examinations, and can be obtained at the time of initial presentation or during early follow-up of patients with pituitary adenomas. This enables the model to be feasibly applied for early risk assessment and stratification of PA. By integrating multiple predictors into a visualized nomogram, the model provides clinicians with a structured and individualized risk prediction tool, facilitating the early identification of pituitary adenoma patients at high risk for PA. Based on this risk stratification, clinicians may tailor follow-up intensity, optimize perioperative monitoring strategies, and, when appropriate, adjust therapeutic approaches or surgical timing, thereby allowing more intensive clinical management of high-risk patients before the occurrence of apoplexy.

The combination of meta-analysis and the nomogram model is a key strength of this study. By integrating evidence from previous literature with clinical data from our center, we provided a more systematic assessment of risk factors for PA. The meta-analysis summarized risk factors reported in existing studies, while the nomogram was developed using patient-level data and internally validated, demonstrating good stability, interpretability, discrimination, and calibration (44). The two approaches complement each other, enhancing the reliability and clinical value of the risk assessment. Nevertheless, several limitations should be acknowledged. First, the retrospective cohort design may introduce unavoidable selection bias, such as inconsistent imaging sources and tumor size measurement methods across patients, which may affect the results. Second, the relatively small sample size may reduce statistical power and limit the generalizability of the findings. Third, molecular or genetic markers, which may provide additional predictive value, were not included in the present study.

Finally, although the nomogram showed good internal performance, its clinical application should be interpreted in conjunction with comprehensive clinical judgment. External validation and model recalibration in multicenter, prospective cohorts are still required to further evaluate its clinical utility for early risk prediction and management decision support in PA.

## 5 Conclusions

This study confirmed through meta-analysis that non-functioning pituitary adenoma, male sex, and hypertension are associated with the occurrence of PA. Based on a single-center cohort, we developed and validated a nomogram prediction model incorporating tumor functional status, sex, hypertension, anticoagulant therapy, and prolactin level. The model demonstrated good discrimination and calibration performance, and can be used to identify high-risk patients. Clinically, enhanced monitoring and early intervention for high-risk individuals are warranted to reduce the occurrence of apoplexy and related adverse outcomes.

## Data Availability

The original contributions presented in the study are included in the article/Supplementary material, further inquiries can be directed to the corresponding authors.
